# EnsembleSE: identification of super-enhancers based on ensemble learning

**DOI:** 10.1093/bfgp/elaf003

**Published:** 2025-04-19

**Authors:** Wenying He, Jialu Xu, Yun Zuo, Yude Bai, Fei Guo

**Affiliations:** School of Artificial Intelligence, Hebei University of Technology, No. 5340, Xiping Road, Beichen District, Tianjin 300400, China; Hebei Province Key Laboratory of Big Data Calculation, Hebei University of Technology, No. 5340, Xiping Road, Beichen District, Tianjin 300130, China; School of Artificial Intelligence, Hebei University of Technology, No. 5340, Xiping Road, Beichen District, Tianjin 300400, China; School of Artificial Intelligence and Computer Science, Jiangnan University, No. 1800 Lihu Avenue, Wuxi 214000, China; School of Software, Tiangong University, No. 399 Binshui West Road, Xiqing District, Tianjin 300387, China; School of Computer Science and Engineering, Central South University, No. 932 South Lushan Road, Changsha 410083, China

**Keywords:** super-enhancer, DNA2vec, ensemble learning, deep learning

## Abstract

Super-enhancers (SEs) are typically located in the regulatory regions of genes, driving high-level gene expression. Identifying SEs is crucial for a deeper understanding of gene regulatory networks, disease mechanisms, and the development and physiological processes of organisms, thus exerting a profound impact on research and applications in the life sciences field. Traditional experimental methods for identifying SEs are costly and time-consuming. Existing methods for predicting SEs based solely on sequence data use deep learning for feature representation and have achieved good results. However, they overlook biological features related to physicochemical properties, leading to low interpretability. Additionally, the complex model structure often requires extensive labeled data for training, which limits their further application in biological data. In this paper, we integrate the strengths of different models and proposes an ensemble model based on an integration strategy to enhance the model’s generalization ability. It designs a multi-angle feature representation method that combines local structure and global information to extract high-dimensional abstract relationships and key low-dimensional biological features from sequences. This enhances the effectiveness and interpretability of the model’s input features, providing technical support for discovering cell-specific and species-specific patterns of SEs. We evaluated the performance on both mouse and human datasets using five metrics, including area under the receiver operating characteristic curve accuracy, and others. Compared to the latest models, EnsembleSE achieved an average improvement of 4.5% in F1 score and an average improvement of 8.05% in recall, demonstrating the robustness and adaptability of the model on a unified test set. Source codes are available at https://github.com/2103374200/EnsembleSE-main.

## Introduction

Gene transcriptional dysregulation, which is one of the core tenets of cancer development, involves in noncoding regulatory elements, including transcription factors (TFs) [1], promoters [2, 3], enhancers [4], super-enhancers (SEs) [5], and ribonucleic acid (RNA) polymerase II (Pol II) [6]. In particular, SEs have been found to play core roles in promoting oncogenic transcription to accelerate cancer progression [7–10]. For instance, cancer-specific SEs have been demonstrated to be key drivers of oncogenic activity in tumor cells [11–14]. Furthermore, it has been confirmed that cancer-specific SEs can mediate the dysregulation of signaling pathways and promote cancer cell growth. Additionally, therapeutic strategies directly targeting SE components, for example, by disrupting SE structure or inhibiting SE cofactors, have shown a good curative effect on various cancers.

Due to the significant role of SEs in various biological processes, precise identification of gene regulatory elements is crucial for biological research and development. SEs were initially identified using enrichment signals of genome-wide MED1 or master TF ChIP-seq (chromatin immunoprecipitation) [15, 16], but the most commonly used marker for identifying SEs is the histone modification H3K27ac [17, 18]. The Rose program developed by the Young group is typically used for bioinformatics-based identification of SEs, where multiple enhancers identified by MED1/master TF or H3K27ac within a specific genomic distance are stitched together and further ranked based on their ChIP-seq signals. Despite many successful experimental methods for identifying SEs, traditional experimental methods [19–21] for identifying SEs still have challenges and significant limitations. Firstly, conducting ChIP analysis to map SEs in all known tissues and cell types is time-consuming and labor-intensive [22]. Secondly, the specificity of antibodies targeting SE-related markers may be lacking in certain cell types, limiting the further application of experimental methods. Its limitations generally manifest in complex experimental design, long experimental cycles, significant experimental investment, and the inability to be scaled up for large-scale applications.

With the advancement of statistical learning and computational biology, an increasing number of researchers are utilizing machine learning and deep learning techniques to analyze large-scale genomic data for the identification and prediction of SEs. To date, there are two main approaches that employ machine learning algorithms to computationally recognize SEs or their constituents (individual enhancers within an SE). The Improse method published by Khan and Zhang [23] integrates 32 ChIP-seq features and three sequence features to predict SEs and their constituents using six different machine learning models. Another approach, named DEEPSEN [24], incorporates 36 features and employs convolutional neural networks to identify the constituents of SEs. The majority of features in both methods rely on costly and time-consuming wet-lab experimental data, leading to challenges when experimental data is limited, despite the easy accessibility of deoxyribonucleic acid sequence information, preventing genomic-level application. In 2021, a deep learning model named DeepSE proposed by Ji *et al.* [25]. addressed this issue. The model predicts SEs solely based on sequence feature embedding. Specifically, it uses DNA2vec [26] to statically encode DNA sequences into unified dimensional embedding vectors, followed by the use of convolutional neural networks to extract features for classification. In 2023, Luo *et al.* introduced the SENet method [27], which optimizes the oversight of DeepSE’s use of DNA2vec by neglecting positional information of nucleotides before and after the coding. By integrating convolution, attention pooling, and transformers, SENet constructs a deep learning network that further enhances model performance. However, both methods primarily consider the abstract semantic relationships of sequences and overlook some biological features composed of specific nucleotides, such as physicochemical properties. Additionally, due to the inherent characteristics of deep learning, the internal weights and parameters are often intractable, and the learned features may be highly abstract and nonlinear. This makes it challenging to provide a clear interpretation of these features, leading to poor generalizability.

Addressing the limitations of previous work, we introduce EnsembleSE, a novel model that incorporates a feature design strategy integrating deep and shallow features, as well as local structures and global information. The architecture utilizes deep learning and machine learning to separately classify deep and shallow features, with the predictions integrated before the output layer using a soft voting strategy to determine the final classification. To validate its effectiveness, we conducted independent testing on datasets for human and mouse species, cross-cell tests, and performance comparisons with existing methods. In the context of the human dataset, EnsembleSE has demonstrated a superior overall performance over both DeepSE and SENet, with its area under the receiver operating characteristic curve (AUROC) notably surpassing the threshold of 0.8 for the first time. On the mouse dataset, EnsembleSE has achieved an average performance lead of 2.3% over DeepSE and SENet, particularly excelling in the metrics of F1 and recall (Rec), thereby reflecting its robust overall performance. In the task of SE identification, EnsembleSE stands as the best model. Notably, our model pioneers the representation of sequences from a biological feature standpoint and innovates feature representation by focusing on information density across different regions of the sequence through segmentation. This approach significantly bolsters the model’s interpretability, offering new insights and a framework for future research in the field.

## Materials and methods

### Datasets

**Figure 1:**
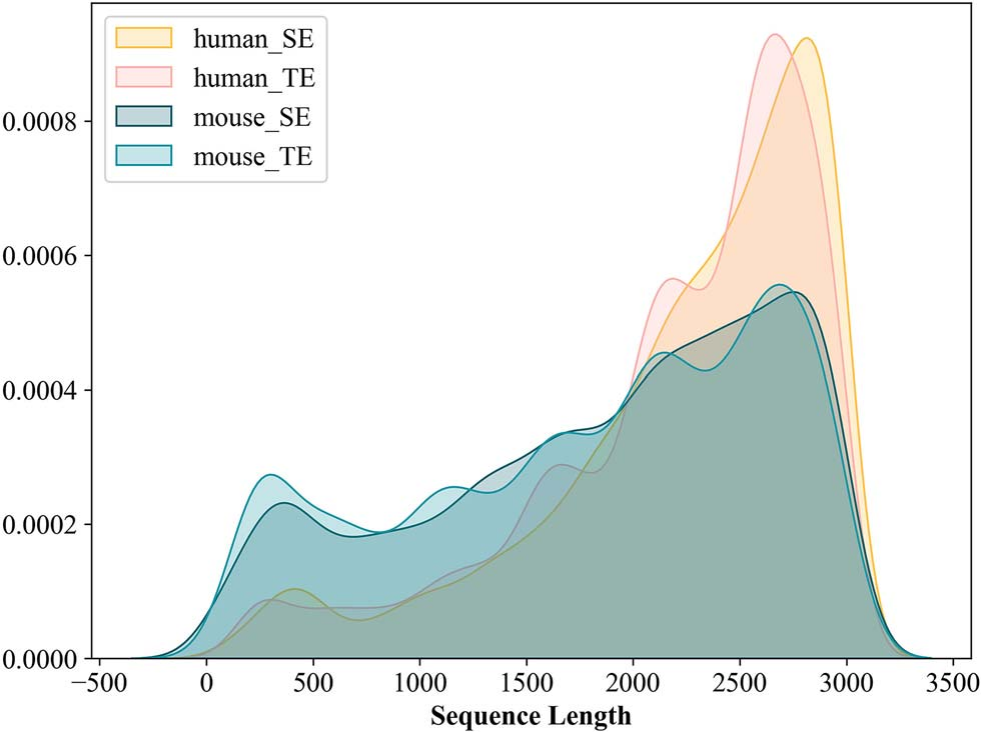
The Kernel Density Estimation of TEs and SEs.

The mouse and human genomic datasets used in this study were obtained from Luo *et al.* [28], based on SEdb2.0 [29](http://www.licpathway.net/sedb/). The datasets consist of training and independent testing sets for both mouse and human genomes. During the model construction process, we used the hold-out method for model evaluation, with 80% of the training set allocated for training and 20% for validation. The sequences in these datasets range in length from 0 to 3000 base pairs (Fig. 1). SEs were considered as positive samples, whereas typical enhancers (TEs) were considered as negative samples.

### Feature representation integrating local structure and global information

Considering the length of the sequences, encoding the entire sequence based on frequency records such as k-mers counts may result in the loss of local information, which may not effectively represent the entire sequence. Therefore, in all encoding methods used in this experiment, the sequences were first segmented before encoding. Specifically, each sequence to be represented was divided into three equal segments: upstream sequence, midstream sequence, and downstream sequence. These three segments were then encoded using the same feature representation method separately and concatenated afterwards.

#### Tri-segment DNA2vec

As an important pre-trained language model, DNA2vec is a vital tool for decoding the complexities of the genomic language. It learns vector representations by considering the contextual information of k-mers, which helps to capture local dependencies and patterns within the sequences. Our training data for word embedding includes the mouse genome (mm9) and the human genome (hg19) obtained from the UCSC Genome Browser. We used these genome files to train DNA2vec, resulting in two 64-dimensional word embedding matrices with k-mers ranging from 3 to 6 (AAA, …, TTTTTT). For each sequence segment of length L, the sequence is divided using a sliding window of size k with a step size of 1, thereby extracting L-k + 1 k-mer segments. The corresponding k-mers vectors were retrieved from the pre-trained embedding matrix, and then the 64-dimensional vectors corresponding to the L-k + 1 k-mers were summed up.


(1) \begin{equation*} {U}_{deep}^j=\sum \limits_{i=0}^{L-k+1}w\left(L\left(i:i+k\right)\right),j\in \left(1,2,3\right) \end{equation*}

(2) \begin{equation*} {V}_{deep}=\left[{U}_{deep}^1,{U}_{deep}^2,{U}_{deep}^3\right]\qquad\qquad\quad\\ \end{equation*}

where$k$is the fixed k-mers length, $l$is the sequence length, $L\left(i:i+k\right)$ represents the k-mers nucleotides on the sequence, and $w(kmer)$is the k-mers vector corresponding to the trained word embedding matrix, $j\in \left(1,2,3\right)$indicates the sequence positions, corresponding to the upstream, midstream, and downstream regions of the DNA sample, respectively.

#### Tri-segment 3-mer composition

- mer representation is widely used to represent gene sequences. In this study, feature vectors were constructed by investigating the frequency of occurrence of consecutive substrings of length 3 in the sequence, as well as their distribution in the upstream, midstream, and downstream regions. Specifically, the sequence was divided into three segments, and for each segment, the frequency of occurrence of all possible combinations of 3-mer nucleotides was calculated. This method is computationally simple yet information-rich, and it can reflect the underlying regulatory motif features in the sequence. The formula is as follows:

(3) \begin{equation*} {U}_{3 mer\_ freq}^j=\left[{f^j}_{AAA},{f^j}_{AAC},...,{f^j}_{TTT}\right],j\in \left(1,2,3\right) \end{equation*}

(4) \begin{equation*} {V}_{3 mer\_ freq}=\left[{U}_{3 mer\_ freq}^1,{U}_{3 mer\_ freq}^2,{U}_{3 mer\_ freq}^3\right]\quad\ \ \end{equation*}

$f$ represents the frequency feature of 3-mer nucleotides, where ${f}_{AAA}^j,{f}_{AAC}^j,...,{f}_{TTT}^j$ denote the occurrence frequencies of trinucleotide combinations in specific sequences.$j\in \left(1,2,3\right)$corresponds to the upstream, midstream, and downstream regions of the DNA sample.

#### Tri-segment PseEIIP

Nair *et al.* [30] utilized a set of electronic-ion interaction pseudo potential (EIIP) indices to numerically represent the four nucleotides A, G, C, and T in DNA sequences. The efficacy of the EIIP numerical method has $V=\left[{U}^1,{U}^2,{U}^3\right]$ been demonstrated through studies such as the prediction of the 5-exon gene F56F11.4, the identification of the cystic fibrosis gene, and the recognition of enhancers [31–33]. EIIP_A_ to EIIP_C_ presents the EIIP values for the four nucleotides, where EIIP_A_ = 0.1260, EIIP_T_ = 0.1335, EIIP_G_ = 0.0806, and EIIP_C_ = 0.1340. In this paper, we construct feature vectors using the average EIIP values of A, T, G, and C in DNA sequence samples, with the formula as follows:


(5) \begin{equation*} {U}_{pseEIIP}^j=\left[ EII{P_{AAA}}^{\ast }{f}_{AAA}^j, EII{P_{AAC}}^{\ast }{f}_{AAC}^j,..., EII{P_{TTT}}^{\ast }{f}_{TTT}^j\right],j\in \left(1,2,3\right) \end{equation*}

(6) \begin{equation*} {V}_{pseEIIP}=\left[{U}_{pseEIIP}^1,{U}_{pseEIIP}^2,{U}_{pseEIIP}^3\right]\qquad\qquad\qquad\qquad\qquad\qquad\\ \end{equation*}

In the formula, $EII{P}_{AAA}, EII{P}_{AAC},..., EII{P}_{TTT}$represent the EIIP values for 3-mer nucleotides, and$f$denotes the frequency of 3-mer nucleotides, with$j\in \left(1,2,3\right)$indicating the upstream, midstream, and downstream regions of the DNA sequence, respectively.

### Feature fusion

In this study, we employed a feature-level fusion strategy that combines feature vectors from different perspectives into a unified feature representation through direct concatenation. Our approach involves learning both deep features extracted through word embedding methods and shallow features derived from physicochemical properties. For deep feature fusion, we integrated features from 4-mer, 5-mer, and 6-mer methods, which have been demonstrated in previous studies to be effective k-mer feature extraction approaches for super-enhancer tasks. This integration resulted in a 576-dimensional feature vector. For shallow feature fusion, we combined 3-mer composition features and pseEIIP features, each initially 192-dimensional, yielding a 384-dimensional fused feature vector.

### Feature selection

We extract two types of feature: semantic vectors from word embedding and biological vectors from physicochemical properties. Word embedding features are used directly in deep learning models to learn complex data relationships during training. The semantic vectors’ high dimensionality and complexity preclude traditional feature selection, so feature selection is only applied to the biological features. We propose using the F-score method for selecting an optimal feature subset that correlates highly with the target variable, thus simplifying the feature space and avoiding overfitting. The F-score value of the *j*th feature is defined as follows:


(7) \begin{equation*} F-\mathrm{score}(j)=\frac{{\left({\overline{x_i}}^{\left(+\right)}-\overline{x_i}\right)}^2+{\left({\overline{x_i}}^{\left(-\right)}-\overline{x_i}\right)}^2}{\frac{1}{n^{+}-1}\sum \limits_{k=1}^{n^{+}}{\left({x}_{k,j}^{\left(+\right)}-{\overline{x_i}}^{\left(+\right)}\right)}^2-\frac{1}{n^{-}-1}\sum \limits_{k=1}^{n^{-}}{\left({x}_{k,j}^{\left(-\right)}-{\overline{x_i}}^{\left(-\right)}\right)}^2} \end{equation*}

In the formula, ${\overline{x_i}}^{\left(+\right)}$and ${\overline{x_i}}^{\left(-\right)}$represent the average value of the *i*th feature in the SE and TE datasets, respectively. ${n}^{+}$represents the number of SEs in the training samples, ${n}^{-}$represents the number of TEs in the training samples, ${x}_{k,j}^{\left(+\right)}$ represents the value of the *i*th feature in the *k*th SE sample, and ${x}_{k,j}^{\left(-\right)}$ represents the value of the *i*th feature in the *k*th TE sample. Obviously, the larger the F-score value, the stronger the discriminative ability of the feature.

### Multi-model ensemble strategy

**Figure 2:**
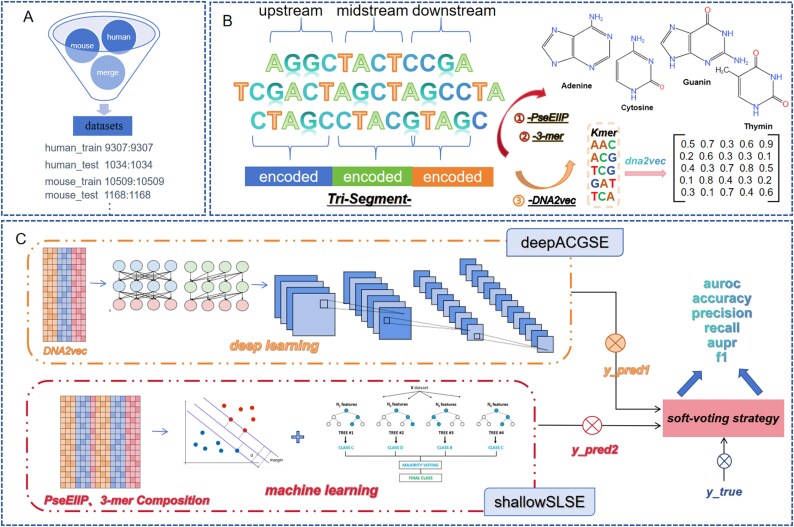
The architecture of our proposed EnsembleSE framework. Panel (a) shows the collection of positive and negative sample datasets for SE, which includes balanced datasets of mouse, human, and a mix of mouse and human data. (b) Multi-level feature processing module—first, the different regions of the sequence are segmented, then deep features based on deep learning and shallow features based on biological low-dimensional characteristics are extracted separately and fused together, resulting in an efficient representation method for the samples. (c) Classification module—integrating the binary classification probability values from both deep learning and machine learning models to make predictions.

Biological sequences possess a rich hierarchical structure and information content. Ensemble models can leverage this information more comprehensively to enhance classification performance. Therefore, this paper constructs a high-precision ensemble model named EnsembleSE, which is based on a deep learning model, deepACGSE (A deep learning model utilizing an extraction method based on word embedding.), and multiple machine learning models, shallowSLSE (a machine learning model based on biological low-dimensional shallow features.). These models utilize deep and shallow features for classification through deep learning and machine learning algorithms, respectively. The predicted data are integrated before the output layer, and the final prediction is given using a soft voting strategy to complete the classification task. The framework of the EnsembleSE model is illustrated in Fig. 2.

#### deepACGSE: deep learning module based on deep features

**Figure 3:**
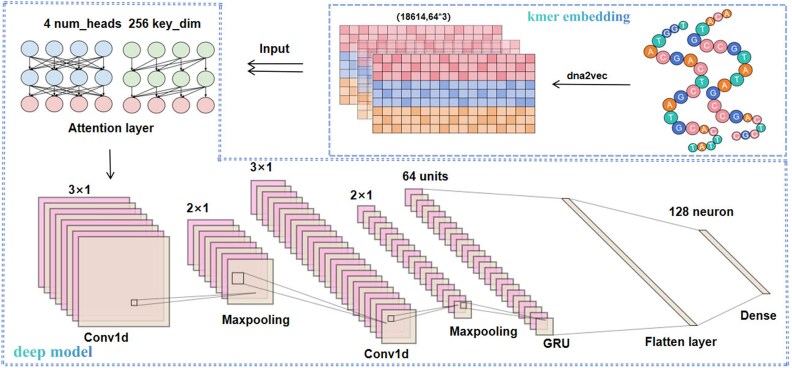
The overall architecture of deepACGSE.

Firstly, we constructed a deep learning model. The architecture of deep learning models allows them to learn representations of data at hierarchical and abstract levels. This capability makes deep learning particularly adept at learning features based on data. Features represented by word embedding contain more abstract data relationships, and theoretically, the characteristics of deep learning models can better capture the complex relationships within data. Therefore, we built a deep neural network for classification based on the deep features represented by word embedding. Specifically, the constructed deep learning model includes components such as multi-head attention layers, convolutional neural network (CNN) layers, max pooling layers, gated recurrent units (GRU) layers, flatten layers, fully connected layers, and the output layer. The detail architecture of deepACGSE as shown in Fig. 3.

The multi-head attention layer enhances the model’s comprehension of input sequences by detecting correlations and focusing on relevant information. CNN layers capture local patterns through convolutions at various scales, and max pooling layers condense feature maps, preserving key information while reducing parameter count and complexity. GRU layers, following convolution and pooling stages, improve on traditional RNNs by better handling long-term dependencies. Dropout after GRU aims to prevent overfitting and enhance generalization. A Flatten layer prepares data for further model processing, and fully connected layers lead to the output layer, delivering the model’s predictions.

#### shallowSLSE: machine learning module based on shallow features

Traditional machine learning algorithms often produce models that are more interpretable, especially when using biological low-dimensional features extracted based on physical or chemical properties. Each feature in such cases corresponds to a specific attribute or characteristic, making it easier to relate these features to actual biological processes and mechanisms. Therefore, the predictive outcomes of the models are more understandable and interpretable. In contrast to features extracted by word embedding, features based on physicochemical properties are discrete, and they typically offer faster computational speeds compared to continuous features. Hence, to achieve more efficient classification, reduce computational complexity, and facilitate the understanding and identification of key factors in SE recognition, we employ machine learning algorithms to recognize biological low-dimensional features extracted based on physical and chemical properties.

In this study, we selected the best-performing combination of six machine learning models (SVM, LightGBM, XGBoost, GBDT, RF, and ExtraTree) for classifying TEs and SEs. The selection of each model was based on its performance on the super-enhancer dataset. SVM was chosen for its ability to find the optimal hyperplane for classifying different classes. LightGBM and XGBoost, as representatives of gradient boosting frameworks, are known for their strong feature learning capabilities and computational efficiency. GBDT builds strong classifiers through iterative training, while RF and ExtraTree improve classification stability and generalization by constructing multiple decision trees and using a voting mechanism. Ultimately, we selected the optimal model combination to ensure both accuracy (ACC) and computational efficiency in the classification task. The choice and combination of models rely not only on the performance of individual models but also on the consideration of data sparsity, scale, and noise. By capturing the patterns and features of the data from different perspectives, the combined strengths of the models are leveraged to ensure the accurate identification of enhancers and SEs.

#### Ensemble strategy

In this section, we integrate the predictions from deep learning and machine learning methods based on a soft voting strategy. Unlike hard voting, which only considers the final predicted labels, soft voting makes more informative decisions by utilizing the probability predictions from each base classifier. Specifically, each classifier provides probability estimates for both classes, and the final prediction is obtained by averaging the probabilities across all classifiers. This can be expressed using the following formula:


(9) \begin{equation*} p\left(y|x\right)=\frac{1}{N}\sum \limits_{i=1}^N{p}_i\left(y|x\right) \end{equation*}

In this equation, $p\left(y|x\right)$ represents the final probability prediction of the ensemble model for the input, $N$ denotes the number of base classifiers, and ${P}_i\left(y|x\right)$ represents the probability prediction of the *i*th classifier.

### Evaluation metrics

Utilizing a variety of metrics allows for a comprehensive evaluation of the model’s performance. Therefore, we have employed five different metrics to assess the predictive performance of EnsembleSE: these include ACC, Precision (Pre), Rec, F1, and the AUROC. The area under the curve (AUC) is defined as the area under the ROC curve, which is calculated by plotting the false positive (FP) rate against the true positive (TP) rate. Meanwhile, To comprehensively address the influence of data partitioning randomness on model performance, all experiments in this chapter were evaluated through multiple repetitions. ACC, Pre, and other metrics are defined as follows:


(10) \begin{equation*} \left\{\begin{array}{l} ACC=\frac{TP+ TN}{TP+ TN+ FP+ FN}\\[4pt] {} Precision=\frac{TP}{TP+ FP}\\[4pt] {} Recall=\frac{TP}{TP+ FN}\\[4pt] {}F1=\frac{2\times TP}{2\times TP+ FP+ FN}\end{array}\right. \end{equation*}

Where TP refers to the number of samples correctly predicted as positive, true negative refers to the number of samples correctly predicted as negative, FP refers to the number of negative samples incorrectly predicted as positive, and false negative refers to the number of positive samples incorrectly predicted as negative by the model.

## Result

### Analysis of feature fusion

Feature fusion enhances model performance by combining diverse feature types. This section discusses the fusion strategy of two types of features. The goal is to find the best fusion approach in both deep learning and machine learning modules, evaluating their effectiveness in improving classification model robustness through comparative experiments.

(1) Effectiveness analysis of deep feature fusion

**Figure 4:**
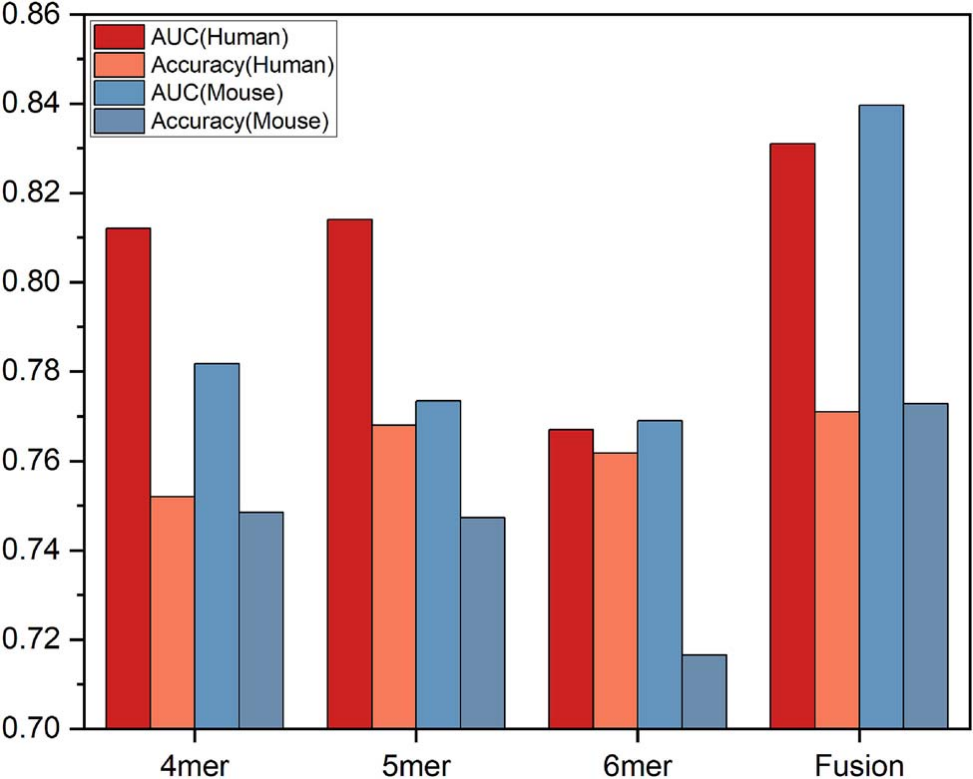
The performance of tri-segment word embedding features of different k-mers on mouse and human datasets.

To verify the effectiveness of the fusion of tri-segment word embedding features of different k-mers, we used SVM as the baseline classifier to compare the fused fusion features with the unfused 4-mer, 5-mer, and 6-mer word embedding features on both mouse and human datasets. The comparison results, as shown in Fig. 4, clearly indicate that the Fusion combination outperforms the individual k-mer combinations across all metrics. For instance, on the human dataset, the AUC value of Fusion is close to 0.84, significantly higher than that of 4-mer at 0.81, 5-mer at 0.82, and 6-mer at 0.77; the ACC is also close to 0.78, higher than all other single combinations. On the mouse dataset, the AUC and ACC of Fusion are above 0.84 and 0.77, respectively, also superior to other single combinations. It can be seen that the method of fusing different k-mer combinations significantly enhances the classification performance of the model.

(2) Effectiveness analysis of shallow feature fusion

**Figure 5:**
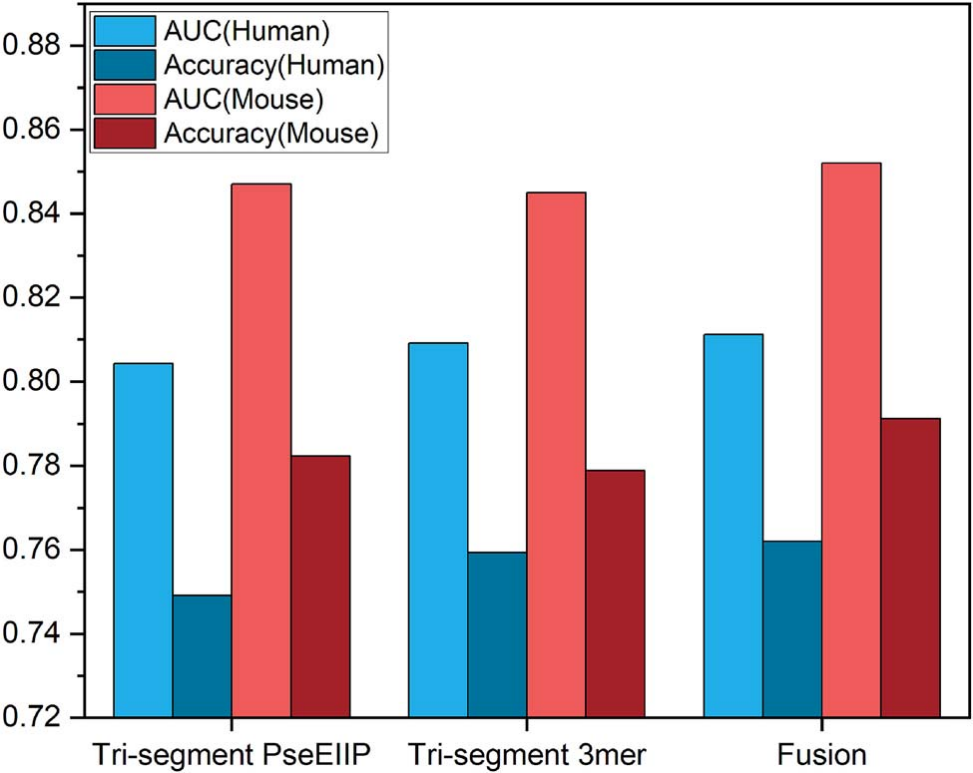
The performance before and after the fusion of different shallow features.

To verify the effectiveness of the fusion of two biologically effective low-dimensional features, Tri-segment PseEIIP and Tri-Segment 3-mer Composition, we used SVM as the baseline classifier to compare the fused fusion features with the unfused features on both mouse and human datasets. The comparison results, as shown in Fig. 5, indicate that the Fusion combination outperforms the individual features of Tri-segment PseEIIP and Tri-segment 3-mer in all metrics. For example, on the human dataset, the AUC value of Fusion is above 0.81, and the ACC is above 0.76, higher than other single combinations, indicating that fusion enriches the feature representation of the data. On the mouse dataset, the AUC and ACC of Fusion are approximately 0.85 and 0.79, respectively, also superior to other single combinations. This demonstrates the advantage of the fusion method in enhancing the performance of the model.

### Experimental setup for feature selection

Considering the characteristics of the feature representation methods and their practical significance, we conducted F-score feature selection for the two types of features represented from the biological perspective of sequences and physicochemical properties, aiming to effectively reduce dimensionality. We selected 80% of the data training set as the training set for feature selection and 20% as the test set for feature selection. During the feature selection process, we used the SVM as the classifier and the F-score method as the experimental technique. In the stepwise feature selection, k denotes the number of features added in each iteration. For both the PseEIIP and 3-mer features, which are both 192-dimensional, they were re-sorted based on the F-score values from highest to lowest. We then increased the number of selected features by increments of k and observed the trend in ACC on the test set. In the experiment, k was set to 10.

**Figure 6:**
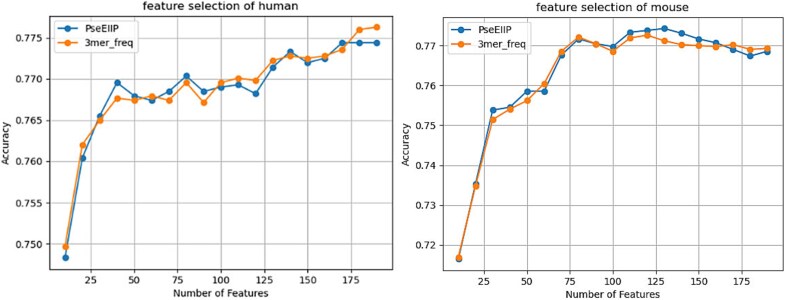
ACC of selecting different feature dimensions on SVM. Panels (a) and (b) represent the ACC at different feature dimensions on human and mouse datasets, respectively.

Figure 6 shows the ACC of two feature representation methods across different dimensions for mouse and human datasets. On the human dataset, ACC increased with more features and fluctuated, suggesting no redundancy and that the full 192-dimensional features are necessary. On the mouse dataset, ACC peaked between 120 to 130 dimensions and then declined with further increases. Thus, we chose the top 120 dimensions by F-score as the final feature set for both methods.

### Model building process

Firstly, we selected the base classifiers in the shallow model and conducted performance comparison experiments for machine learning classifiers. We selected 80% of the training data from the dataset as the training set for this experiment, and the remaining 20% as the test set. We compared six machine learning classifiers on the dataset represented based on Tri-Segment PseEIIP and Tri-Segment 3-mer Composition (as shown in the Table 1). The top 4 accuracies are marked in bold, corresponding to the classifiers LightGBM, XGBoost, GBDT, and SVM.

**Table 1:** Performance comparison of different machine learning methods on the human and mouse datasets

Species	Model	AUC	ACC	AUPR	F1	Rec	Pre
Human	LightGBM	0.850	**0.776**	0.854	0.774	0.765	0.783
	XGBoost	0.842	**0.769**	0.846	0.767	0.761	0.774
	RF	0.829	0.763	0.832	0.759	0.746	0.772
	ExtraTree	0.825	0.758	0.828	0.753	0.738	0.769
	GBDT	0.839	**0.770**	0.843	0.766	0.752	0.781
	SVM	0.836	**0.776**	0.826	0.773	0.764	0.783
Mouse	LightGBM	0.845	**0.770**	0.862	0.753	0.700	0.814
	XGBoost	0.830	**0.765**	0.851	0.754	0.719	0.791
	RF	0.820	0.748	0.834	0.726	0.667	0.796
	ExtraTree	0.817	0.734	0.832	0.705	0.636	0.791
	GBDT	0.832	**0.757**	0.848	0.737	0.679	0.805
	SVM	0.831	**0.773**	0.839	0.754	0.697	0.822

Note: The top 4 accuracies for each species are shown in bold.

Subsequently, we selected these four classifiers - D, L, S, G, and X (representing deepACGSE, LightGBM, SVM, GBDT, and XGBoost, respectively) - to integrate with deep learning classifiers. As described in Section 2.5.3, the goal was to maximize ACC on the training set and provide data support for constructing an overall ensemble model. As shown in Table 2, it can be observed that the best overall performance is achieved when integrating three models on both human and mouse datasets. Particularly, on the mouse dataset, the ACC exceeds 0.78 after integrating deep learning, SVM, and LightGBM, significantly surpassing other ensemble combinations. Therefore, we conclude that our final model is the ensemble model consisting of a deep learning model and two traditional machine learning models: SVM and LightGBM. Concurrently, we also compared the performance of the shallow feature module (shallowSLSE) and the deep feature module (deepACGSE) prior to integration, with detailed information provided in Supplementary Table S1.

**Table 2:** Performance comparison of various classification combinations

Species	Methods	AUC	ACC	AUPR	F1	Rec	Pre
Human	D + L	0.847	0.778	0.848	0.775	0.764	0.786
	D + L + S	0.860	**0.786**	0.862	0.781	0.768	0.798
	D + L + S + G	0.845	0.776	0.842	0.770	0.752	0.790
	D + L + S + G + X	0.859	0.785	0.857	0.784	0.779	0.789
Mouse	D + S	0.843	0.778	0.855	0.760	0.702	0.827
	D + S + L	0.846	**0.781**	0.865	0.765	0.712	0.827
	D + S + L + X	0.850	0.777	0.859	0.760	0.707	0.822
	D + S + L + X + G	0.844	0.773	0.859	0.754	0.697	0.823

### Comparison of EnsembleSE with the existing methods

To validate the EnsembleSE model’s effectiveness in identifying SEs, we compared it with existing methods, focusing on DeepSE and SENet, which also use sequence data. We followed SENet’s evaluation protocol for consistency. Since DeepSE utilized an imbalanced dataset and employed SMOTE and random undersampling, we omitted these for a fair comparison. We assessed all models using five metrics on identical test datasets across two species. The comparative results are detailed in Table 3.

**Table 3:** Performance comparison with existing methods on the human and mouse datasets

Species	Model	AUC	ACC	F1	Rec	Pre
Human	DeepSE	0.772	0.743	0.727	0.856	0.633
	SENet	0.797	0.740	0.737	0.729	0.745
	EnsembleSE	**0.807**	**0.746**	**0.751**	0.765	0.737
Mouse	DeepSE	0.808	0.803	0.725	0.670	0.790
	SENet	0.842	0.846	0.704	0.609	0.836
	EnsembleSE	0.833	0.782	**0.771**	**0.734**	0.812

The bolded metrics in Table 3 highlight the indicators where EnsembleSE achieved significant improvements compared to the other two models. Specifically, on the human dataset, EnsembleSE outperformed existing methods across all evaluation metrics. Notably, the model achieved an AUC of 0.807 and F1 score of 0.751, exceeding the previous best results of 0.8 and 0.75, respectively. This demonstrates that the model exhibits enhanced overall performance in the task of super-enhancer identification. Particularly on the mouse dataset, the F1 score achieved 0.771, markedly surpassing both DeepSE and SENet, indicating the robustness of species-specific genetic analysis. We have explored and validated the performance of the EnsembleSE model on cross-species data, conducting extensive experimental verifications on both mouse and human datasets, with detailed information available in the Supplementary file, Figure S1.

## Discussion

### Segmental specificity analysis of trinucleotide composition

**Figure 7:**
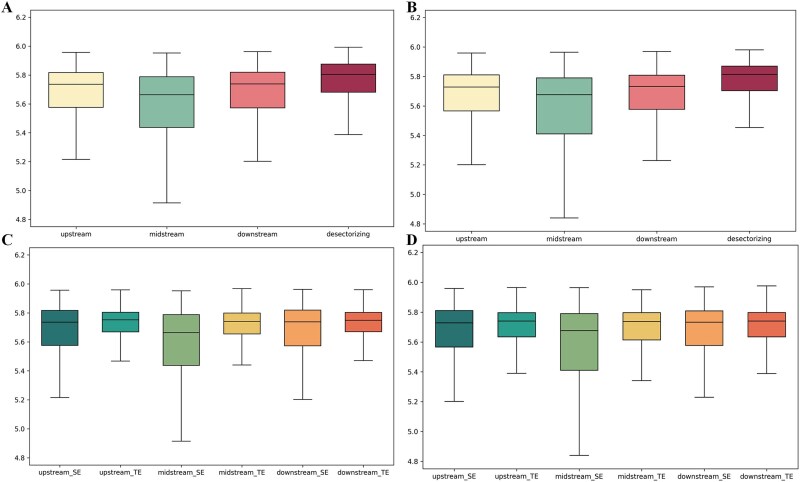
Box plot of entropy for the upstream, midstream, downstream, and overall sequence. Panels (a) and (b) are the box plots of entropy distributions for different segments of samples in human and mouse datasets, respectively. Panels (c) and (d) are the box plots of entropy distributions for different segments of positive and negative samples in human and mouse datasets, respectively.

We initially analyzed the entropy of trinucleotide compositions in whole and segmented sequences, using box plots to illustrate information content differences, as shown in Figs. 7a–d. The segmented approach provides a clearer view of feature contributions across sequence regions and scales. Firstly, the difference in boxplots between segmented and unsegmented data indicates that segmenting allows for a better understanding of the feature contributions in different regions of the sequence and enables the analysis of sequence features at different scales. Secondly, by observing the distribution of positive and negative samples in each segment, we found differences in the data distribution of TEs and SEs in each region, further confirming that the features we extracted are key factors in identifying SEs.

### Interpretability analysis of shallow features based on SHAP

**Figure 8:**
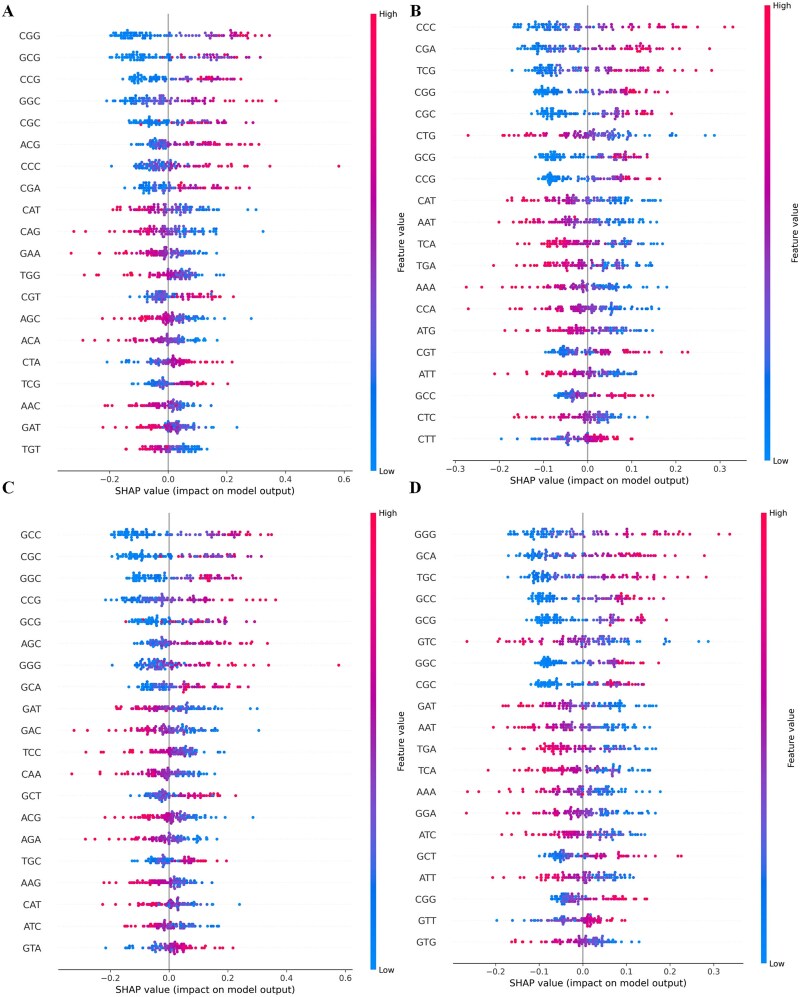
SHAP Plot for shallow feature. Panels (a) and (b) represent the SHAP plots for the Tri-Segment PseEIIP in mouse and human datasets, respectively. Panels (c) and (d) are the SHAP plots for the Tri-Segment 3-mer Composition in mouse and human datasets, respectively.

SHAP plots are used to visualize and explain the contribution of each feature in model predictions to the final outcome. If certain features appear frequently and have a significant impact in the SHAP plots, it usually indicates that these features play an important role in the model’s predictions. Figure 8a and b describes the importance of EIIP features in identifying SEs in mouse and human cells, respectively. Figure 8c and d describes the importance of 3mer features in identifying SEs in mouse and human cells. By comparing the SHAP plots on the mouse and human datasets, we can observe that the positive and negative impacts of 3mer nucleotide combinations on identifying SEs are quite similar within the same species, but the impact varies greatly between different species. At the same time, by observing the red and blue sample points on the SHAP plots, it can be seen that the biologically effective low-dimensional features we extracted based on 3mer segments show a regular distribution in the task of identifying SEs, that is, a certain 3mer combination has almost a positive or almost a negative impact on performance, further indicating that our features represent the data well and are effective.

### Visualization analysis of deep embedding features based on principal component analysis

**Figure 9:**
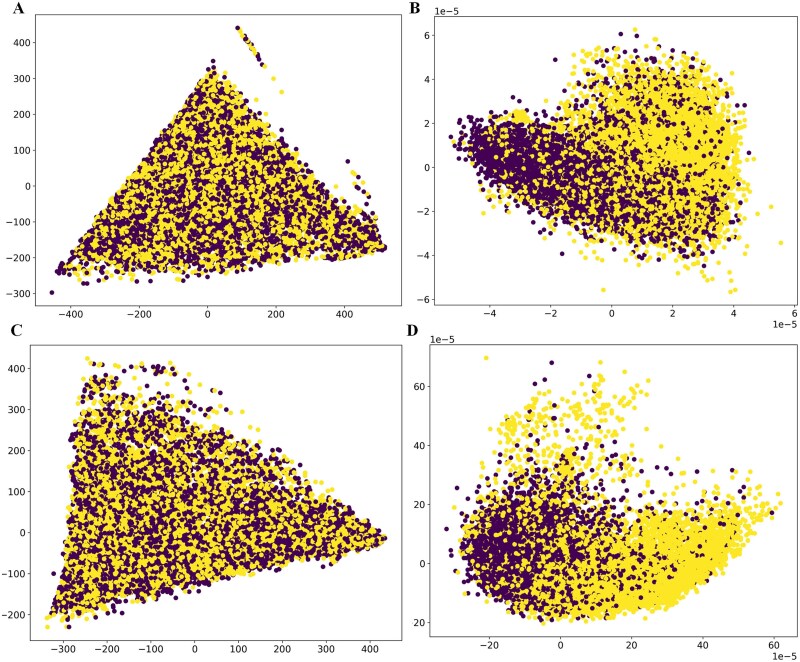
PCA visualization of feature space distribution in deep learning. (a) Feature space of the output of deepACGSE embedding layer on human species. (b) Feature space of the output of deepACGSE transformer layer on human species. (c) Feature space of the output of deepACGSE embedding layer on mouse species. (d) Feature space of the output of deepACGSE transformer layer on mouse species.

In this section, to gain a better understanding of how our constructed deep learning model learns effective representations, we utilize principal component analysis (PCA) technique to visualize the spatial distribution of SEs and TEs across different species, as depicted in Fig. 9. In the figure, each point represents a DNA sequence, with different colors distinguishing between positive and negative samples (yellow circles denote positive samples, while purple circles denote negative samples). Our research findings indicate that in the original sequence feature space formed by DNA2vec extraction, the distribution of positive and negative samples is completely mixed together, making it difficult to distinguish. However, features learned after convolutional pooling operations and GRU layers show two relatively distinct clusters, with positive and negative samples clearly separated in the feature space. These results suggest that our constructed deep model can effectively extract latent features of sequences encoded using word embedding. In summary, this section validates that the deep model component of our EnsembleSE model can accurately distinguish between SEs and TEs based solely on sequence information.

## Conclusion

In this paper, we introduce EnsembleSE, a novel ensemble learning framework designed for identifying SEs within transcription enhancers (TEs) using sequence data alone. In developing the classification model, we leverage the strengths of both deep learning and traditional machine learning approaches. EnsembleSE effectively integrates both shallow and deep sequence features, capturing local and global information. Additionally, the use of segmented feature extraction methods combined with low-dimensional biological features provides a fresh perspective for exploring and identifying SEs. Looking ahead, several avenues could further advance this research. First, the current framework could be enhanced by incorporating additional biological features, such as chromatin accessibility data and transcription factor binding sites, which may provide valuable supplementary information for SE identification. Second, exploring transfer learning techniques could improve the model’s ability to generalize across different cell types and species, broadening its practical applicability. From a computational standpoint, future work could focus on statistical methods and interpretable biological shallow features to further identify key DNA segments or sequence positions that contribute significantly to SE recognition, offering valuable insights into the biological mechanisms behind SE function. Furthermore, investigating semi-supervised learning methods would enable the use of abundant unlabeled genomic sequence data, potentially improving model performance even with limited labeled data. These advancements would not only improve the ACC and reliability of SE identification but also deepen our understanding of gene regulatory mechanisms, ultimately paving the way for more effective therapeutic strategies.

**Key Points**

- Employs a multi-perspective feature extraction, integrating local and global sequence data for clearer biological pattern recognition.
- Combines multi-model insights for superior generalization, enhancing predictive performance on diverse datasets.
- EnsembleSE achieves significant improvements in F1 score, evidencing robustness in species-specific genetic analysis.

## Supplementary Material

Supplementary_File_BFGP-24-0242_elaf003

## Data Availability

The dataset and code for EnsembleSE are available at https://github.com/2103374200/EnsembleSE-main.
