# Supplementary material for: EnsembleSE: identification of super-enhancers based on ensemble learning
Source: Brief Funct Genomics. 2025 Apr 19;24:elaf003. doi: 10.1093/bfgp/elaf003 (PMC12008123; doi:10.1093/bfgp/elaf003)
Supplement: Supplementary_File_BFGP-24-0242_elaf003 [file supplementary_file_bfgp-24-0242_elaf003.docx]

**Supplementary Information**

## Cross-species validation

This section aims to explore and validate the capability of our proposed model EnsembleSE in identifying super-enhancers within typical enhancers across cross-cell-line datasets. Given that typical enhancers and super-enhancers may exhibit variations in different cell lines, cross-cell-line validation becomes a crucial step in assessing the model's generalization performance. Therefore, we conducted extensive experimental validations on both mouse and human datasets to evaluate the robustness and reliability of the model across different species and cell lines. Through this cross-cell-line validation, our goal is to comprehensively understand the applicability of the EnsembleSE model in various cellular environments, providing strong support for further elucidating the role of super-enhancers in gene regulation.

To provide a clearer demonstration of the performance of the ensemble model and its individual components in cross-cell-line testing, we not only generated results for EnsembleSE but also for the deep model using only word embedding features, as well as for the two machine learning classifiers utilizing biological low-dimensional shallow features. These results are presented in **Figure S1**.


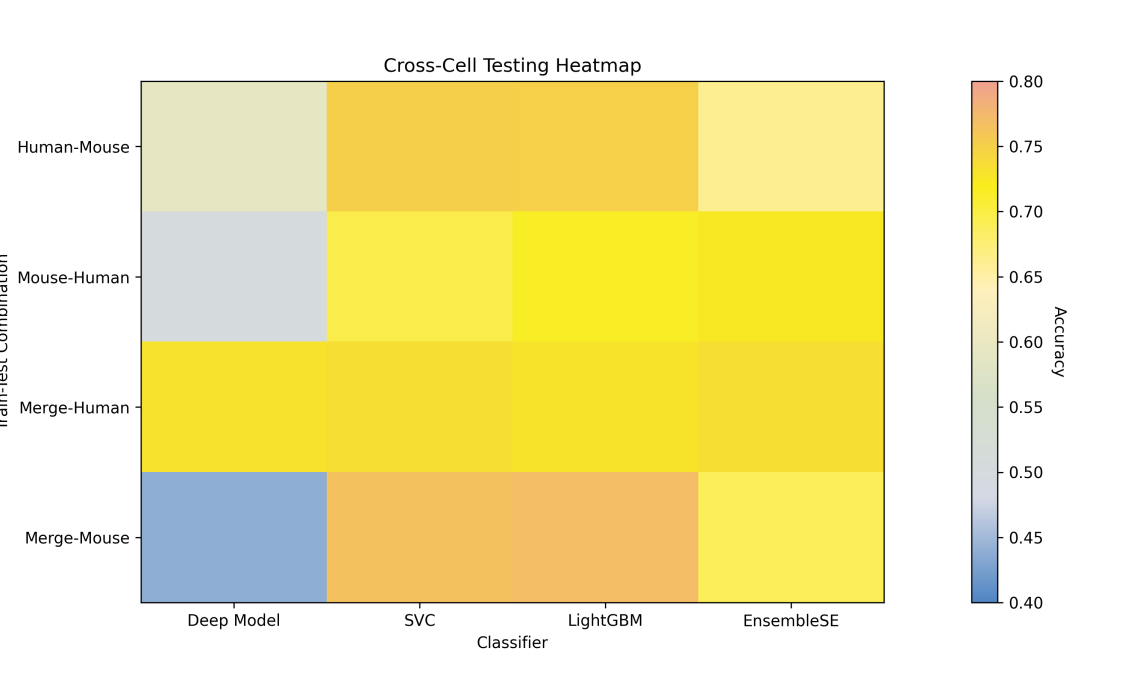


**Figure S1.** The heat map of the accuracy value performing the cross-cell line validation. The vertical axis represents the cell lines used for training the model and the corresponding test performance cell lines, and the horizontal axis indicates the local and global models of the ensemble model.

We observed that EnsembleSE did not perform as expected in cross-species testing. Further analysis revealed that, in the deep learning model part, aside from the accuracy being around 0.7 when testing the human dataset using a mixed dataset of mouse and human, the predicted accuracy in other cases was around 0.5. This suggests that the poor performance of the deep learning model affected the overall performance of the final ensemble model. To explore the reasons for the poor performance of cross-species testing in the deep learning model, we consulted literature and found that super enhancers exhibit specificity, meaning they have different sequence patterns in different cell types, which may affect the performance of cross-cell-line testing. However, in machine learning classifiers, cross-species testing achieved good results. Comparative analysis suggests that this may be because the word embedding feature method based on deep learning mainly relies on the textual features and contextual information of the data. Although it performs well on the training data, due to the specificity of different cell types, its generalization to unseen cell types is poor. In addition to this, word embedding methods capture general features and may not accurately capture the biological differences between different cell types, resulting in lower accuracy and stability in predictive tasks across cell types.

Relative to word embedding-based representations, the bio low-dimensional feature representation method based on physicochemical properties may have several advantages that lead to better results in cross-cell-line testing. First, they offer stronger biological interpretability. For instance, the PseEIIP feature in this study directly reflects the electronic ion interaction potential of biomolecules, while the Tri-Segment 3-mer Composition feature reflects the distribution of different k-mer nucleotides in various regions, both of which are closely related to biological functions. Hence, they are more likely to maintain a certain degree of consistency across different cell lines. Second, physicochemical-based features generally exhibit higher stability and repeatability. Compared to word embedding-based features, they are less influenced by the dataset or training algorithm, making them more suitable for maintaining consistent performance in cross-cell-line scenarios. Third, bio low-dimensional features typically have lower dimensions, making them easier to transfer and apply across different cell lines, thereby reducing the likelihood of decreased generalization performance due to the curse of dimensionality. In summary, although our work did not achieve the expected results in cross-cell-line validation at the overall model level, our work provides a promising direction for exploring the identification of super enhancers within typical enhancers in cross-species testing, particularly in terms of local models and features.

**Comparison with different components**

**Table S1.** Performance on the human and mouse datasets, compared with different components

| Species | Model | AUC | ACC | AUPR | F1 | Recall | Precision |
| --- | --- | --- | --- | --- | --- | --- | --- |
| Human | deepACGSE | 0.832 | 0.763 | 0.834 | 0.756 | 0.726 | 0.789 |
|  | shallowSLSE | 0.847 | 0.775 | 0.852 | 0.773 | 0.756 | 0.791 |
|  | EnsembleSE | **0.860** | **0.786** | **0.862** | **0.781** | **0.768** | **0.798** |
| Mouse | deepACGSE | 0.832 | 0.768 | 0.850 | 0.758 | **0.715** | **0.840** |
|  | shallowSLSE | **0.851** | 0.776 | 0.864 | 0.761 | 0.699 | 0.834 |
|  | EnsembleSE | 0.846 | **0.781** | **0.865** | **0.765** | 0.712 | 0.827 |
